# Attitudes towards urban howler monkeys (*Alouatta caraya*) in Paraguay

**DOI:** 10.1007/s10329-022-00975-5

**Published:** 2022-02-10

**Authors:** Marco Alesci, Rebecca L. Smith, Jorge Damian Ayala Santacruz, Andrea Camperio Ciani

**Affiliations:** 1 Department of Philosophy, Sociology, Pedagogy, and Applied Psychology, University of Padova, Padua, Italy; 2 Fundación Para La Tierra, Centro IDEAL, 321, Mariscal José Félix Estigarribia, c/Teniente Capurro, Pilar, Ñeembucú Paraguay; 3 School of Biological Sciences, University of Aberdeen, Tillydrone Ave, Aberdeen, UK

**Keywords:** Ethno-primatology, South America, Local perceptions, Semi-structured interviews, Urban primates

## Abstract

Increasing urbanisation is encroaching into natural habitats and sometimes forcing wildlife into urban centres. Whether or not wildlife can thrive in an urban environment is dependent on many factors, one of which is how the species is perceived by local people. This study focuses on the city of Pilar in south-west Paraguay, which is home to a population of urban-dwelling black and gold howler monkeys (*Alouatta caraya*). Using semi-structured interviews, we assessed peoples’ attitudes towards the presence of howlers, whether they perceived the monkeys to cause problems in the city, what they believed were the biggest threats to the monkeys, and whether they felt that the presence of monkeys in the city was compatible with their way of life in the long term. Overall, we found that the majority of interviewees had positive attitudes towards the monkeys, believing that they brought benefits to the city and that they should be protected from potential risks in the urban environment. Our results provide the basis for collaborative, community-based development of management strategies for the long-term survival of these urban monkeys.

**Supplementary Information:**

The online version contains supplementary material available at 10.1007/s10329-022-00975-5.

## Introduction

The study of the human/non-human primate (hereafter primates) interface, ethno-primatology, is a multidisciplinary field that recognises humans as a natural part of ecosystems (Fuentes 2012; Setchell et al. 2017). When studying wild primates using an ethno-primatological approach, the anthropogenic realities of the modern world are included as integral factors affecting all aspects of the ecology and behaviour of wild primates (Malone et al. 2014). With 60% of primate species now threatened with extinction as a result of human activities (Estrada et al. 2017), it is essential to consider the social and cultural aspects of conservation issues to successfully implement effective, long-term solutions (Setchell et al. 2017).

Around 55% of the world’s human population lives in urban centres, with this expected to rise to 68% by 2050 (United Nations 2018). Continuing urbanisation represents a major threat to wildlife through the destruction and fragmentation of natural habitats (McKinney 2006). In some areas, loss of natural habitats results in wildlife, including some species of primates, utilising the urban environment (Sinha and Vijayakrishnan 2017). Cities present several unique opportunities for primates, including abundant and rich (though often non-natural) food resources and the absence of natural predators (Sinha and Vijayakrishnan 2017). However, the urban environment also presents various novel threats to individual survival, such as electrocution on power lines and attacks by domestic dogs (*Trachypithecus vetulus nesto,* Moore et al. 2010), road kills (*Alouatta guariba clamitans*, Teixeira et al. 2013; *Alouatta caraya*, Para La Tierra unpublished data), novel gastrointestinal parasitic infections (*Papio anubis*, Ryan et al. 2012) and direct conflict with humans (Lee and Priston 2005).

Human–wildlife conflict has costs for both humans and wildlife. Direct costs for humans can include physical injuries, disease transmission, property damage, livestock depredation and crop losses, while indirect costs could include cultural dilemmas, restricted movement, increased fear or stress, and needing to guard personal belongings (Hockings and Humle 2009; Humle and Hill 2016; Madden 2004). Baboons (*Papio*), macaques (*Macaca*) and vervet monkeys (*Chlorocebus*) raid crops throughout Africa and Asia, and in many areas have become established in urban centres (Lee and Priston 2005). In cities, the high concentration of food resources can lead to primates raiding houses, shops or garbage heaps (Sinha and Vijayakrishnan 2017). This food provisioning (whether intentional or not) can over-habituate primates to human presence, potentially increasing the likelihood of aggressive interactions (Lee and Priston 2005; Sinha and Vijayakrishnan 2017) and opportunities for disease transfer. Consequently, primates living in close proximity to humans are sometimes considered “pests” and are the target of aggressive actions (Lee and Priston 2005; Schilaci et al. 2010). It is important to understand attitudes towards wild primates at a local level, as attitudes and behaviour vary according to cultural and traditional differences (Lee and Priston 2005; Schilaci et al. 2010).

In several parts of Central and South America, howlers (*Alouatta*) appear to adjust well to living in urban environments where relationships with humans are positive (*Alouatta guariba clamitans,* Buss et al. 2015; Chaves and Bicca-Marques 2017; *Alouatta pigra*, Alexander 2000; *Alouatta palliata,* Valenzuela-Córdova et al. 2015). To date, no studies have examined the relationship between humans and Paraguay’s only species of *Alouatta*, the black and gold (or Paraguayan) howler (*A. caraya*).

The black and gold howler is widespread through central to southern South America, being found in Paraguay, Brazil, Bolivia, Argentina and possibly Uruguay (Bicca-Marques et al. 2020; Jardim et al. 2020). Although black and gold howlers primarily inhabit seasonally dry semi-deciduous and deciduous forests (Bicca-Marques et al. 2020), they can thrive in disturbed habitats and forest fragments in agricultural areas or human settlements (Crockett 1998; di Bitetti et al. 1994; Horwich 1998; Johns and Skorupa 1987; Muhle 2008). Despite their wide distribution and environmental adaptability, black and gold howlers are considered Near Threatened due to population decline, habitat loss, hunting pressure and susceptibility to disease outbreaks (e.g., yellow fever) (Bicca-Marques et al. 2020).

**Fig. 1:**
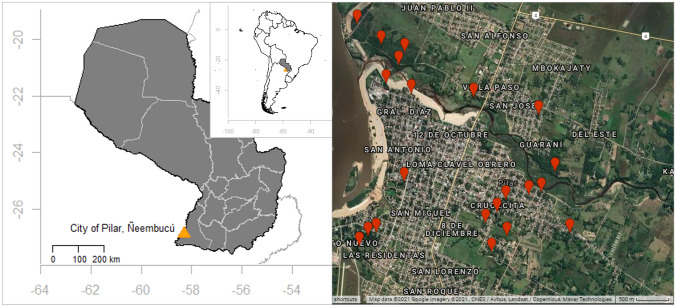
Location of Paraguay in South America, the city of Pilar in Paraguay, and the locations of known howler groups inside Pilar from August to December 2017 (Google Earth)

The city of Pilar in Ñeembucú, south-west Paraguay, is home to a population (around 69–100 individuals, Para La Tierra, unpublished data, Fig. 1) of black and gold howlers. These monkeys live in people’s private gardens, in the trees that line the streets, or on the roofs of houses, rather than in public parks or forest fragments, making it crucial to understand people’s attitudes towards them. Any conservation measures must take into account the needs of both people and the monkeys. In this study we evaluated local attitudes towards the howler population in Pilar.

## Methods

### Ethics statement

Our research was approved by the Ministerio de Ambiente y Desarollo Sostenible (MADES), Fundación Para La Tierra and the ethics board of the Department of Philosophy, Sociology, Pedagogy, and Applied Psychology at the University of Padova. The research complied with all local laws. Informed verbal consent was obtained for each interview in accordance with Paraguayan laws and standards (it is not culturally appropriate to ask for written consent). Interviewees’ privacy has been protected by ensuring confidentiality of the data and anonymity. Participants were given the option of completing the interview in Spanish or Guaraní and were debriefed following the interview. All interviewees remained anonymous.

### Study site

The study was carried out in the city of Pilar (26° 51′ 31.5″ S 58° 18.383′ W), the capital of Ñeembucú department in south-west Paraguay (Fig. 1), and home to around 30,000 people (www.dgeec.gov.py/). Its climate is humid, with a mean annual temperature of 22 °C. In summer, temperatures can reach as high as 40 °C, dropping to 2 °C in winter. October–March are the hottest months, while April–September are colder. Rainfall is usually highest in the months of January, March, April and October (National Oceanic and Atmospheric Administration 2015). Pilar is situated in the Ñeembucú Wetland Complex, a naturally mosaic habitat of swamps, humid Chaco gallery forest and grasslands. It is unknown whether the population of monkeys inside Pilar (~ 15 groups with ~ 69–100 individuals) is completely isolated from howlers in the Wetland Complex outside the city.

### Data collection

We conducted 261 interviews between August and December 2017. We developed a pilot interview of 45 questions following the Participatory Sustainability Framework (Camperio Ciani 2010; Camperio Ciani et al*.* unpublished data). The pilot interview was written in English, translated to Spanish by a native speaker and validated by author Ayala Santacruz (a Paraguayan citizen fluent in both Spanish and Guaraní). We interviewed 21 residents between the 16th and 29th August 2017 to test the effectiveness of the pilot questions, and then made minor modifications for the final interview. The final semi-structured interview started with an identification task, in which participants chose primate species they believed they had personally observed in Pilar (and that they thought were present in other parts of Paraguay) from several pictures of native and non-native species. As black and gold howlers are sexually dichromatic, two pictures (one male and one female) were shown to the interviewees, as it is common for people in Paraguay to believe that the two sexes are two separate species (Smith, pers. obs.)

Data from participants who chose non-native species were included in the analyses if they demonstrated awareness of the urban howler groups, as this reflected failure to recognise the howlers from the pictures rather than lack of knowledge about their presence in the city. The interview consisted of 31 questions (19 open, 12 closed: Supplementary Table 1) covering five principal domains, three questions on sociodemographic information (age, sex, length of residency in Pilar) and two filter questions to direct the interview:

1. Description of their encounters (six questions): participants were asked to describe their encounters with the howlers, where they had seen them, the behaviour they had observed, peoples’ behaviour towards the monkeys and the monkeys’ responses to people.

2. Perceived costs and benefits (five questions): we asked participants to list the costs and benefits to them or the community resulting from the presence of monkeys. When a cost was identified, we asked the participants to evaluate its severity based on its impact on the community or the participant’s life. We also asked what hazards howlers faced in Pilar and how serious participants believed them to be.

3. Attitudes towards the urban howlers (three questions): participants were asked to describe how they felt about the howlers, using a free-listing exercise (Smith, 1993; Smith and Borgatti, 1997).

4. Awareness of urban howlers (seven questions): participants were asked about their knowledge of howler ecology and behaviour and the number of howler groups in Pilar, and whether they were aware of any laws protecting the howlers.

5. Compatibility, reversibility and future expectations (five questions): participants were asked their opinions on whether the coexistence with the urban howlers is compatible with people’s present lifestyle and whether the situation might change in the future.

At the beginning of the interview we introduced ourselves and the purpose of the research, explicitly stating that it was aimed at understanding the interviewee’s point of view and that there were no right or wrong answers. We conducted interviews every Friday and Saturday from September to December 2017 in Spanish or Guaraní, according to the interviewee’s preference. Interviews were carried out in eight neighbourhoods throughout the city, each of which had at least one resident howler group. We used non-random sampling to include people who lived within or adjacent to howler home ranges, as they were likely to encounter the monkeys more frequently (Table 1). In addition to interviewing people in their neighbourhoods, we interviewed employees of the Pilar Cotton Factory, Ministry of the Environment (MADEs), the Pilar Municipality, and the Administración Nacional de Electricidad (ANDE). Interviews lasted between 10 and 20 min, with the semi-structured format allowing adjustments to questions if needed. At the end, the interviewee was fully debriefed. As not all interviewees answered every question, the number of responses to each question varied (Supplementary Table 1).

**Table 1:** Distribution of interviews throughout different neighbourhoods in Pilar

Neighbourhood	Number of interviewees	% of total interviewees
Barrio Obrero	42	16
Barrio General Díaz	43	16
Barrio Villa Paso	32	12
Barrio Crucecita A*	40	15
Barrio Crucecita B*	33	13
Barrio San Antonio	33	13
Barrio Las Residentas	6	2
Barrio San José	32	12
Total	261	100

*More interviews were conducted in Barrio Crucecita, as two separate howler groups live there. As their home ranges do not overlap (Para la Tierra, unpublished data), the households affected by their presence are different

### Sociodemographic information

Of the interviewees, 145 were male and 116 were female, with ages ranging from 18 to 86 years (44 ± 16, mean ± SD). Among them, 208 (83%) had lived in Pilar all their life or for more than 15 years, 40 (16%) for less than 15 years, and three (1%) only occasionally visited the city. These data were not available for 10 interviews. We interviewed between 32 and 75 people in each neighbourhood, with the exception of Barrio Las Residentas, where only six people were interviewed (Table 1).

### Data analysis

Statistical analysis was carried out using Microsoft Excel 2013 and SPSS version 23 software. Chi-squared analyses were used to determine whether interviewees who encountered the howlers in their gardens were more likely to report problems caused by them than interviewees who encountered the monkeys around the city. Similarly, we compared the number of people in each neighbourhood who reported issues, to assess whether some howler groups caused more problems than others. A two-sided Fisher exact test was used to examine whether interviewees’ perceptions of the severity of damage caused by the monkeys influenced their attitudes towards their presence. The free-listing exercise was analysed using ANTHROPAC 4.0 (Borgatti 1996). The salience of each adjective was calculated using the number of mentions and its position in participants’ lists. Adjectives that were widely used and listed at the start of the lists were considered most salient. The salience index can range from 0 (item never mentioned) to 1 (item mentioned by all participants and always first on the list) (Borgatti 1996), but as we did not have an expected list of adjectives, a score of zero was not possible. Plural and singular declensions of items (as well as their synonyms) were aggregated into the same answer category. Separated adjectives with slightly different connotations were kept to preserve the original meaning.

## Results

### Pre-interview species identification task

A total of 191 interviewees recognised monkeys of both sexes (73%), whereas 45 (17%) recognised the adult female only and 19 (7%) recognised the adult male only. Only four interviewees (2%) were not aware of the presence of howlers in the city (Table 2).

**Table 2:** Responses to the species identification exercise (both sexes of *Alouatta caraya* are included because their different colours can lead to people believing they are two separate species)

Species	Present in Pilar	Present in Paraguay	Percentage (*N* = 260) who believed the species was present in Pilar
Adult male howler (*Alouatta caraya*)	Yes	Yes	81%
Adult female howler (*Alouatta caraya*)	Yes	Yes	91%
Hooded capuchin (*Sapajus cay*)	This species does not occur naturally in Ñeembucú but people do keep it as a pet in Pilar	Yes	8%
Chacoan titi monkey (*Plecturocebus pallescens*)	No	Yes	4%
Azara’s owl monkey (*Aoutus azarae*)	No	Yes	1%
Black-tailed marmoset (*Mico melanurus*)	No	Yes	0.4%
Sumatran orangutan (*Pongo abelii*)	No	No	4%
Olive baboon *Papio anubis*	No	No	0.4%
Ring-tailed lemur *Lemur catta*	No	No	0.4%

Interviewees were asked to identify what species they believed to be present in Pilar and in other parts of Paraguay from a series of photographs

### Semi-structured interviews

#### Description of human–howler encounters

Regarding encounters with howlers, 236 of 261 (90%) interviewees had seen howlers in Pilar during the previous month, and a further 19 (7%) had seen monkeys at some point in the past. Of the 236 people who had seen the monkeys within the previous month, 125 (48%) encountered monkeys more than three times in a week. In describing their encounters with the howlers, 216 interviewees (86%) evaluated them as “peaceful”, 32 (13%) considered some interactions to be “neutral” and nine (4%) labelled some interactions as “aggressive”.

Howlers were seen on trees by 252 participants (99%), and 116 interviewees often saw them in their private back gardens (45%), 56 on rooftops (22%) and 51 on power lines (22%). Most respondents (255, 89%) reported that howlers tended to ignore their presence, and simply observing the monkeys was their most common response (192 interviewees, 75%) (Table 3 a–d).

**Table 3:** Responses to where interviewees had seen howlers, the different monkey behaviours they had observed, peoples’ behaviour towards the monkeys and the monkeys’ responses to people

Question	Answer 1	Answer 2	Answer 3	Answer 4	Answer 5	Answer 6
a. Interviewees who reported seeing the monkeys in different locations						
Where have you see the monkeys?	In trees	In my garden	On the ground	On rooftops	On power lines	
Number of responses	252	116	65	56	50	
b. What behaviours the interviewees who had seen the monkeys had observed						
What have you seen the monkeys doing?	Feeding	Resting	Howling	Travelling		
Number of responses	140	68	57	40		
c. How interviewees who had seen the monkeys responded to them						
What did you do when you saw the monkeys?	Watched them	Took photographs	Fed them	Ignored them	Threw rocks or chased them	Chased them
Number of responses	192	32	29	21	11	9
d. How the monkeys responded to interviewees who had observed them						
What did the monkeys do when you saw them?	Ignored me	Self-scratching or hiding (stress- or fear-related behaviours)	Watched me	Approached and showed their tongues	Aggressive displays (branch shaking)	Made physical contact
Number of responses	228	47	43	7	4	1 (possibly an ex-pet)

Interviewees could give multiple responses to each question

Of the 255 interviewees who had observed the monkeys, 251 described how they felt during encounters. A total of 187 interviewees (75%) reported positive emotions, 53 (21%) had no feelings towards them and 11 (4%) reported negative emotions.

### Perceived costs and benefits of howler presence

Overall, the presence of the howlers in the city did not cause issues for people, with 241 interviewees (93%) reporting that they had “no” or “unimportant” personal issues with the monkeys. Interviewees who reported specific monkey-related problems were asked to rate the severity of the problem (Table 4). The most common problem was monkeys’ faeces in garden (21 interviewees, 8%), although this was usually rated “unimportant” (12, 57%).

**Table 4:** The number of interviewees who reported a specific problem caused by the presence of the monkeys and how they perceived the severity of the issues

Problem	Number of interviewees who reported the issue	Perceived severity of the problem			
		Unimportant (%)	Moderate (%)	Serious (%)	Not specified (%)
Faeces	21	57	24	19	–
Foraging	17	41	18	35	6
Howling	9	44	33	22	
Roof	7	43	14	43	
Urine	2	–	50	50	
Fear	2	–	50	50	
Dogs	1	–	–	100	

According to 105 interviewees (40%), the presence of monkeys had created problems for other local people. Faeces in people’s gardens was again the most commonly reported problem (62 interviewees, 24%), although it was most often rated “unimportant” (35, 56%).

The number of interviewees who reported problems caused by monkeys in their homes was greater than that of those who did not encounter them in their homes (*χ* = 32.58, df = 2*, P* < 0.0001). The number of people reporting damage caused by monkeys did not differ significantly among neighbourhoods (*χ*^2^ = 10.76, df = 6, *P* = 0.096).

When asked whether the presence of the howlers in Pilar provided personal benefits, 170 interviewees (67%) reported that they did, with the most common benefit reported being the intrinsic “beauty” of seeing the monkeys (164 interviewees, 65%). In terms of benefits for the overall community, 209 interviewees (84%) believed that the monkeys’ presence benefited the community, with 79 (32%) believing they could improve ecotourism and 73 (29%) believing that their presence was an important opportunity for children to see wildlife.

The most frequently reported threats to howlers included people’s use of slingshots to scare them away from their gardens (103 interviewees, 42%) and risk of electrocution on power lines (98, 39%). Of the 98 interviewees who reported power lines being an issue, 80 (82%) perceived the risk as “serious” (Table 5).

**Table 5:** The number of interviewees who reported a specific risk the urban environment posed to the monkeys and how they perceived the severity of the risks

Problem	*N*	Number of interviewees who reported the issue	Perceived severity of the problem			
			Unimportant (%)	Moderate (%)	Serious (%)	Not specified (%)
Slingshot/throwing objects	245	103	20	23	55	1
Power lines	252	98	6	12	82	–
Dogs	248	79	29	18	53	–
Killed by people	247	41	2	12	85	–
Others	258	16	19	19	56	6

### Attitudes towards urban howlers

In each neighbourhood surveyed, most interviewees reported a positive attitude towards the monkeys; negative attitudes were less common. More interviewees reported “neutral” or no strong feelings towards the howlers than negative perceptions of them (Table 6).

**Table 6:** Overall attitude towards presence of the urban howlers in different neighbourhoods

Neighbourhood	*N*	Positive (%)	Neutral (%)	Negative (%)
Barrio General Diaz	43	93	7	0
Barrio Obrero	37	84	8	8
Barrio Villa Paso	31	84	16	0
Barrio San José	30	83	10	7
Barrio Crucecita (Group A)*	38	79	16	5
Barrio San Antonio	32	75	22	3
Barrio Crucecita (Group B)*	33	70	15	15
Barrio Las Residentas	6	67	33	0

*Barrio Crucecita hosts two different howler groups (Group A and Group B) whose home ranges do not overlap (Para La Tierra, unpublished data). For this reason, interview data from people living around these groups were analysed independently to assess their attitude towards the specific group

Interviewees who experienced serious monkey-related issues significantly reported less appreciation of their presence in the city (*P* < 0.0001, two-sided Fisher’s exact test). Though only eight people experienced such issues, five of them (63%) also rated their presence in the city as “negative”. In contrast, only six (3%) of the 214 interviewees who believed the howlers did not cause damage reported not liking their presence. Howlers are more often perceived in a positive than negative manner. The notably salient attributes associated with the howlers were “Gentle” (*S* = 0.31), “Beautiful” (*S* = 0.229) and “Good” (*S* = 0.181) (Table 7).

**Table 7:** Results of salience analysis

	Description in Spanish	Translation to English	Frequency (*N* = 253)	Average rank	Salience
1	Manso	Gentle	36	1.36	0.31
2	Lindo	Beautiful	27	1.39	0.229
3	Bueno	Good	21	1.29	0.181
4	Simpático	Funny	6	1.69	0.048
5	Inteligente	Smart	5	1.62	0.039
6	Llamativo	Striking	5	1.85	0.036
7	Pasivo	Inactive	4	1.44	0.028
8	Atractivo	Attractive	4	2.1	0.024
9	Persona	Human-like	3	1.5	0.024
10	Juguetón	Playful	3	1.57	0.022
11	Tierno	Sweet/cute	3	2.13	0.021
12	Ruidoso	Noisy	2	1.33	0.02
…	…	…	…	…	…
24	Perjudicial	Harmful	1	1	0.008
25	Malo	Mean/bad	1	1	0.008

*Frequency* percentage of interviewees that mentioned the term, *Average rank* average position of the item in the participant lists, *Salience* salience index

Few interviewees expressed beliefs or superstitions involving the howlers. Sixteen believed that monkeys bring diseases (7%) (specific diseases were not mentioned), and 11 (5%) commented that humans descended from monkeys. Other less common themes about beliefs and myths involving the howlers are shown in Table 8.

**Table 8:** Percentage of interviewee responses reporting beliefs and myths about howlers in Pilar

Reported belief	Percentage of response (*N* = 228)
Monkeys bring diseases	7
We descended from monkeys	5
If the monkeys howl, tomorrow it will rain	3
If the monkeys defecate on your head, you will lose your hair	2
Monkeys throw their faeces at you	2
Monkeys bring bad luck	1
If you kill a monkey, you will have bad luck	0.4
Monkeys bring good luck if seen moving on the ground	0.4
Monkeys are not harmed by people because they are similar to us	0.4
Monkeys descend from people	0.4

### Awareness of urban howler population and conservation status

When asked about the status of Pilar’s howler population, 110 interviewees (45%) reported that the population size had increased, 55 (23%) believed it had remained constant and 63 (26%) believed it had decreased in recent years. Only 29 interviewees (11%) correctly estimated a population size of more than 60 individuals, while 110 (43%) believed it to be less than 15 individuals. Most interviewees were unaware of the real distribution of the monkeys across the city, with 202 (78%) believing that they were present in only one or two neighbourhoods.

Though 233 interviewees (93%) were aware that hunting howlers is illegal in Pilar, only 135 (54%) knew that it is illegal to keep primates as pets in Paraguay. When asked about consumption of primate meat, 177 interviewees (77%) believed that people in Pilar do not consume primate meat, but 18 (8%) believed that howlers are eaten in Pilar, the surrounding countryside or other Paraguayan cities.

More than half of the interviewees (170: 66%) believed that the howlers face threats to their survival because they live in an urban environment. The most commonly reported threats (interviewees could report more than one threat) were injuries from slingshots (103 interviewees, 42%), electrocution on uninsulated power lines (98 interviewees, 39%), attacks by dogs (79 interviewees, 32%), and 41 interviewees (17%) reported that in Pilar there are people who kill the monkeys (Table 5).

### Compatibility, reversibility and future expectations

The presence of the monkeys was described by 245 interviewees (96%) as compatible with their current lifestyle. However, 183 (73%) believed that the presence of the monkeys is likely to change in the near future, potentially getting worse for monkeys, either as a result of increasing urbanisation (68 interviewees, 27%) or people’s increasingly intolerant behaviour (50, 20%).

When asked about the future of the monkeys in Pilar, 226 interviewees (92%) suggested protecting the howlers, including the creation of a nature reserve (53 interviewees, 22%), more protection through legislation (51, 21%) and the development of a community environmental education program (30, 12%). Concerning responsibility for protecting the howlers, 54 interviewees (23%) believed this fell to the local community, 44 (19%) believed it was the government (MADEs) and 35 (15%) believed the local municipality should be responsible.

## Discussion

We found that the people of Pilar have an overall positive attitude towards the city’s urban howlers. This differs from some studies of urban primates, where it is not uncommon for people to view primates as a threat, either to their safety or to their livelihoods (Hill 2004; Mormile and Hill 2016). The lack of fear of the howlers in Pilar, and the people’s positive perception of their presence, may be related to the monkeys’ behaviour and ecology. Studies in which people describe a negative or fearful attitude towards wild primates tend to concern larger, more terrestrial, or potentially more aggressive species such as baboons (Hill 2004; Mormile and Hill 2016), orangutans (Campbell-Smith et al. 2010), chimpanzees (McLennan 2010), vervet monkeys (Brennan et al. 1985) or macaques (Southwick et al. 2005; Zhao and Deng 1992). Howlers, like all platyrrhines, are mainly arboreal (Back and Bicca-Marques 2019) and in Pilar they rarely descend to the ground. This means that direct encounters between people and the monkeys are uncommon. To our knowledge there are no reported cases of wild howlers attacking humans and the most aggressive behaviour reported by people in Pilar was the shaking of branches. Intragroup aggression is extremely rare in the howlers of Pilar. During a 3-month behavioural study of two groups in 2019, no instances of intragroup aggression were observed (Para La Tierra, unpublished data). This could contribute to the local perception that howlers are calm and peaceful.

In addition to the physical distance between the monkeys and people resulting from the former’s arboreality, howler behaviour is typified by long periods of rest or sleep, often accounting for 60–70% of their time (*Alouatta guariba clamitans*, Back and Bicca-Marques 2019; *Alouatta caraya*, Bicca-Marques and Calegaro-Marques 1994; Overbeck et al*.* in review; *Alouatta pigra,* Pavelka and Knopff 2004). Sleeping high in trees and largely ignoring people means that the monkeys draw relatively little attention of local residents, reducing the likelihood of negative attitudes towards their presence. This situation contrasts with some localities in Africa (Lee and Priston 2005) and Asia (Sinha and Vijayakrishnan 2017), but resembles other places in Central and South America where other *Alouatta* species live in close proximity to humans (*Alouatta guariba clamitans*, Buss et al. 2015; Chaves and Bicca-Marques 2017; *Alouatta pigra,* Alexander 2000).

Many studies of people’s attitudes towards wild primates take place in the context of crop raiding (Campbell-Smith et al. 2010; Freitas et al. 2008; Marchal and Hill 2009; McLennan 2010; Moore et al. 2010). Pilar is a completely urban environment and though many people have small vegetable patches in their gardens, most are not reliant on these crops as a main source of income. Furthermore, the howlers’ arboreality and highly folivorous diet means that groups either do not, or only rarely, forage in people’s gardens (Para La Tierra unpublished data). Similar to the situation with brown howlers (*Alouatta guariba clamitans*, Chaves and Bicca-Marques 2017), local people do not perceive their foraging behaviour as damaging, as the cultivated crops are not exploited commercially. What did cause the monkeys to be perceived as a problem in Pilar was when heavy adults cross people’s roofs, causing damage to the tiles. One potential way to limit this problem in affected areas could be to plant more trees or install rope bridges to provide alternative pathways (Hernández-Pérez 2015; Teixeira et al. 2013), or the creation of a small fund to provide some compensation for the cost of repairing damage caused by the monkeys. Another relatively frequently reported issue was the mess in people’s gardens resulting from fruit dropped by the monkeys as they foraged, or their faeces. Groups of black and gold howlers typically contain between 3 and 18 individuals (Aguiar et al. 2009; Crockett and Einsberg 1987; Rumiz 1990). In Pilar the groups are smaller, ranging from three to nine individuals but usually with only three or four in groups that live mostly in private gardens (Para La Tierra unpublished data), possibly due to the highly fragmented nature of the urban environment or higher mortality than in more natural habitats (though further study is required on this topic). That monkey faeces were not considered a severe issue by many people may be related to the small size of groups in gardens and the resulting small amount of mess. Further study could confirm whether group size is related to the opinions of the people who live closest to the monkeys.

Interviewees were aware of several potential threats that urban life presented to the howler population. The three most commonly reported issues were attacks by domestic dogs, electrocution on uninsulated power lines, and people killing the monkeys. Of these, electrocution was seen as the most serious threat, as the power lines are frequently used for travel by monkeys in several groups, and during the study period, three howler deaths by electrocution were recorded, though fewer such deaths were recorded during subsequent observations on the same groups (Para La Tierra, unpublished data). The electrocution risk could be mitigated by insulating the first 2 m of electric cables, the most dangerous to the monkeys due to the proximity to the transformers, in areas of Pilar with resident monkey groups. This could reduce deaths at a relatively modest cost, as an alternative to the more expensive solution of full insulation of cables, and given that conservation in general is not prioritised by the Paraguayan government. Alongside cable insulation, rope bridges could facilitate monkeys’ travel between disconnected forest patches, reducing their use of power lines or ground passages, as this solution has already proved to be effective for other howler species (*Alouatta pigra*, Hernández-Pérez 2015; *Alouatta guariba clamitans*, Teixeira et al. 2013).

As the majority of people believed that the presence of howlers in the city is compatible with their current lifestyle, and as the monkeys are not expected to cause more problems in the near future, the authors concluded that the situation is possibly sustainable according to the Participatory Sustainability Framework (Camperio Ciani 2010; Camperio Ciani et al*.* unpublished data). Understanding the attitudes of Pilar’s residents towards the howlers is important for planning the long-term conservation of these unique monkeys. As most interviewees are happy with the presence of the monkeys, interventions such as translocations are not necessary in this area. Furthermore, the Ñeembucú Wetland Complex surrounding Pilar already supports very high numbers of howlers (Para La Tierra, unpublished data), is mainly private land and has no protected areas.

People’s positive feelings towards the monkeys suggests opportunities for a more holistic community-driven approach to their conservation, for example, the organisation of cultural and recreational events focused on spreading awareness of primate conservation issues (Franquesa-Soler and Serio-Silva 2017; Franquesa-Soler et al. 2020). Educational events could serve to clarify misconceptions and the consequences of the illegal pet trade for survival of wild populations. Environmental education and community engagement programs are known to be powerful tools in conservation of various primate species in different social and cultural situations (*Cebuella pygmaea*: de la Torre and Yèpez 2003; *Leontopithecus chrysopygus*: Padua 2010; *Nomascus hainanus*: Qian et al. 2021; lemurs (multiple species): Rakotomamonjy et al*.* 2015). In the case of Pilar, these initiatives would provide a platform for open dialogue between stakeholders and provide the foundation for the development of mortality mitigation projects and collaborative conservation measures.

This study clarified local people’s reactions to the presence of wild primates in an urban centre in Paraguay. The monkeys’ arboreal behaviour and long resting periods decrease their direct interactions with humans, making their presence more tolerable. Even when howlers forage in people’s gardens, local attitudes towards them tend to remain positive, as cultivated crops often go undamaged (*Alouatta guariba clamitans*, Chaves and Bicca-Marques 2017) and their presence may not significantly affect people’s lives. In Pilar, although the howlers are appreciated by local people, increasing urbanisation could pose significant threats to their survival by reducing available trees and forcing howlers to travel more frequently on power lines or on the ground. Identifying specific hazards, monitoring attitudes, and implementing and evaluating ad hoc solutions can help to foster long-term, positive howler–human relationships in urban landscapes.

## Supplementary Information

Below is the link to the electronic supplementary material.

Supplementary file1 (DOCX 19 KB)
