# Supplementary material for: Attitudes towards urban howler monkeys (Alouatta caraya) in Paraguay
Source: Primates. 2022 Feb 10;63(2):161–71. doi: 10.1007/s10329-022-00975-5 (PMC8898239; doi:10.1007/s10329-022-00975-5)
Supplement: Supplementary file 1 — Supplementary file1 (DOCX 19 KB) [file 10329_2022_975_MOESM1_ESM.docx]

Supplementary Material: Attitudes towards urban howler monkeys (*Alouatta caraya*) in Paraguay

Supplementary Table 1: Questions of the interview guide and the number of respondents for each of them.

| **Question** | **Number of respondents** | **Domain** | **Type** |
| --- | --- | --- | --- |
| Age | 261 | Sociodemographic | **Open** |
| Gender | 261 | Sociodemographic | **Closed** |
| How long have you been living in Pilar? | 251 | Sociodemographic | Open |
| Identification task | 260 | Awareness of howlers | Closed |
| How often did you see the howlers in the last month? | 261 | Encounter | Closed |
| Most of your encounters with the howlers, were pacific, neutral or aggressive? | 252 | Encounter | Closed |
| Where did you see the howlers? |  | Encounter | Open |
| - On trees | 254 |  |  |
| - On budlings | 253 |  |  |
| - On the ground | 252 |  |  |
| - On power lines | 232 |  |  |
| - In your garden | 255 |  |  |
| When you saw the monkeys, how did they react to your presence? | 255 | Encounter | Open |
| What did you do when you saw the monkeys? | 255 | Encounter | Open |
| How did you feel when you saw the monkeys, positive, neutral or negative? | 251 | Encounter | Closed |
| Do monkeys cause issues to you or your property? If yes, what issues do they cause? | 260 | Cost/Benefit | Open |
| Do monkeys cause issues to the community? If yes, which issues? | 261 | Cost/Benefit | Open |
| Overall, do you have any benefits from the presence of monkeys? | 252 | Cost/Benefit | Open |
| In your opinion, who benefits from the presence of the monkeys? | 248 | Cost/Benefit | Open |
| Are you aware of any danger for monkeys? | 259 | Cost/Benefit | Open |
| - People kill them | 247 |  |  |
| - People throwing objects | 245 |  |  |
| - Power lines | 252 |  |  |
| - Dogs | 248 |  |  |
| - Others | 258 |  |  |
| Overall, how would you evaluate the presence of the monkeys, positive, neutral, or negative? | 254 | Attitude | Closed |
| Free listing exercise - How would you describe these monkeys? | 253 | Attitude | Open |
| What other people think about them? | 228 | Attitude | Open |
| In the last years, howlers' number is decreased, remained the same, or increased? | 243 | Awareness of howlers | Closed |
| How many monkeys there are in the city of Pilar? | 255 | Awareness of howlers | Open |
| Where are they? | 260 | Awareness of howlers | Open |
| Is hunting monkeys legal? | 250 | Awareness of howlers | Closed |
| Is keeping monkeys as pets legal? | 249 | Awareness of howlers | Closed |
| Are you aware if there are people eating the monkeys around here? | 230 | Awareness of howlers | Closed |
| Can you live with the monkeys under the current circumstances? | 251 | Compatibility and Reversability | Closed |
| Do you think that this situation may change in the future? | 251 | Compatibility and Reversability | Closed |
| Why is the situation going to change/not going to change? | 251 | Compatibility and Reversability | Open |
| What would you like to happen in the future in relation to the presence of monkeys? | 245 | Compatibility and Reversability | Open |
| Who would be responsible for managing the situation with the monkeys? | 233 | Compatibility and Reversability | Open |
| Filter question 1 - Have you ever heard of the howlers monkeys (Mono Aullador negro, Manechi negro, o Caraya)? | NA | Filter |  |
| Filter question 2- Have you ever heard of any problems caused by monkeys to the community or to you? Have you ever heard of issues that the howlers have here in Pilar? | NA | Filter |  |
